# Rapid and precise temperature control of samples flowing through a capillary from −20 to 130 °C

**DOI:** 10.1063/5.0304183

**Published:** 2026-02-01

**Authors:** Eli Worth, Valentyn Stadnytskyi, Hyun Sun Cho, Friedrich Schotte, Philip Anfinrud

**Affiliations:** Laboratory of Chemical Physics, NIDDK, NIH, Bethesda, Maryland 20892, USA

## Abstract

The focus of this paper is on the development of an apparatus capable of rapid and precise temperature control of liquid samples circulated through a small-bore glass capillary over a range from −20 to 130 °C, with stability better than 10 mK rms. Temperature regulation is achieved by lowering the capillary into a dry gas stream that flows through a narrow slit in a wide aluminum nozzle whose temperature is controlled by two thermoelectric modules (TEMs) that transfer heat between the nozzle and coolant flowing through a heatsink. Rapid thermal equilibration between the capillary and nozzle occurs primarily through conductive heat exchange with the flowing gas. The performance of the temperature controller is well described by a heat-transfer model, with low heat-capacity components enabling fast temperature slew rates. The temperature-control head is compact and can be fitted with customizable apertures suitable for visual access to the capillary and for transmitting focused electromagnetic radiation through it. However, the thermal cycling of the TEMs at high operating temperatures led to the rapid degradation of their cooling capacity. To mitigate this issue, separate low-(~4 °C), room-, and high-temperature (~85 °C) coolant reservoirs and pinch valves were used to select the coolant reservoir based on the target temperature, thereby limiting the maximum temperature experienced across the TEMs during thermal cycling and dramatically extending their operational lifetime. This apparatus was designed primarily for temperature-dependent, time-resolved x-ray scattering studies of biomolecules in solution but has also been used in time-resolved spectroscopic investigations.

## INTRODUCTION

To generate a more complete, mechanistic understanding of how a biomolecule functions via time-resolved studies, it is beneficial to observe its structural dynamics over a wide range of temperatures spanning from as low as −20 to as high as 130 °C, a range that spans beyond that where organisms can survive, such as arctic fish in subzero water and thermophiles near volcanic vents.^1,2^

A wide range of temperature control methods have been utilized in temperature-dependent studies of biomolecules. Traditional refrigerated water circulators can control temperature with mK precision but offer limited temperatures ranges and slow slew rates.^3^ For example, synchrotron beamlines utilizing thermostatically controlled sample cells and sample exchangers achieve high temperature stability but are typically limited to modest temperature ranges (≈2–60 °C).^4^ To access temperatures lower than 0 °C and above 100 °C, additional precautions are required, such as adding a cryoprotectant to prevent freezing and pressurizing the sample chamber to prevent boiling. Thermoelectric modules (TEMs) have seen widespread use in temperature control systems largely due to their compact size and their ability to heat or cool depending on the sign of the current. Samples in direct thermal contact with TEMs can be controlled over a broad range of temperatures and achieve temperature ramp rates in excess of several degrees per second.^5^ However, geometries that require direct contact with a TEM make it difficult to perform pump–probe time-resolved studies of biomolecule dynamics with x rays. Although TEMs are capable of generating large temperature differences, thermal cycling over the full temperature range accessible is known to cause degradation in TEM performance.^6^

This paper presents a temperature control system capable of rapidly transitioning between temperatures spanning from −20 to 130 °C without harming the TEMs and holding set temperatures with high precision. This system features a novel aluminum gas flow nozzle that facilitates non-contact, convective heat transfer to/from an inserted capillary. An insulated temperature control head precisely controls the temperature of the nozzle and does so quickly, thanks to their small heat capacity. A simple heat transfer equation that takes into account the temperature dependence of TEM thermal conductance is capable of predicting the temperature controller performance with high fidelity over the entire operating range. The origins of the deleterious effects of thermal cycling on TEMs is discussed and mitigated by a multi-reservoir coolant circulation system that limits the temperature across the TEMs, enabling nearly unlimited thermal cycling of the TEMs. This system was designed to non-invasively control the temperature of biomolecule solutions flowing through a small-bore capillary while probing biomolecule structure and dynamics via x-ray scattering. To assess the temperature controller performance, temperature-dependent x-ray scattering of water over a temperature range spanning from −16 to 120 °C is reported. The water scattering signature is strongly temperature dependent and can be used to calibrate the magnitude and time dependence of laser-induced temperature jumps (T-jump) when pursuing time-resolved studies of biomolecule dynamics.

### Aluminum nozzle with laminar gas flow

A lightweight aluminum nozzle was designed to deliver temperature-controlled, laminar gas flow through a slit opening into which a capillary can be inserted (Fig. 1). The gas is introduced through a port in the bottom of the nozzle and equilibrates with the nozzle temperature as it flows from the entrance chamber through an array of 2 × 2 × 0.25-mm channels to the middle chamber. When passing from the middle chamber into the upper exit chamber, the gas passes through a second array of channels, whose gap beyond 2 mm increases linearly up to the knife edge, which slows the gas flow velocity while maintaining laminar flow. Multiple collisions between the gas and nearby wall surfaces when passing through the two sets of narrow channels ensure rapid thermal equilibration. Thanks to its two-piece clam-shell design, it is possible to mill all features needed to create the interleaved arrays of narrow channels with a sturdy 2-mm flat bottom mill bit. Before final assembly, a thin film of thermally conductive grease was “stamped” onto the inner surfaces of each half to minimize the thermal resistance between them.

The laminar gas flow rate out of the nozzle is typically set at 5 cm s^−1^, which consumes gas at a rate of ~0.4 LPM (liters per minute). When a capillary is lowered into the slot of the nozzle, its temperature rapidly equilibrates with the nozzle primarily through convective heat exchange with the gas. Dry gas flowing at this velocity over the capillary suppresses condensation when operating below the ambient dew point. Moreover, the small bore of the capillary limits the volume of solution cooled, and thanks to HF-etching of the capillary interior, ice nucleation is inhibited, which allows aqueous samples to be supercooled to low temperatures without a cryoprotectant. Sample solutions containing biomolecules are routinely supercooled down to −16 °C without freezing.

When pursuing x-ray studies, helium is delivered through the nozzle to reduce x-ray scattering, but for other applications, nitrogen is used. Note that the thermal conductivity of helium is nearly six times that of nitrogen, 0.151 vs 0.026 (W m^−1^ K^−1^), which speeds the rate of temperature equilibration accordingly.

### Temperature control head

The temperature control head and its internal components are shown in Fig. 2. The coolant flows through channels in the 3D-printed heatsinks, which are silicone bonded (Dow 734 Flowable Sealant) to 25 × 25 × 3 mm TEMs (Custom Thermoelectric, Inc., Bishopville, Maryland). Direct contact between coolant and the alumina plates of the TEMs ensures very efficient heat transfer. The temperature of the nozzle is controlled by pumping heat across the TEMs, which are wired in series and in thermal contact with one face of the nozzle via a thin film of thermal joint compound (Boyd Laconia, LLC, Thermalcote I). Since parasitic Joule heating affects TEM efficiency, TEMs with matching resistance are paired to minimize the risk of a temperature gradient from one side of the nozzle to the other.

The nozzle temperature is sensed by two small, fast responding 1.6 × 1.2 × 0.25 mm platinum RTDs (Innovative Sensor Technology, P1K0.161.6W.A.010) wired in series. The RTDs are epoxied into oval apertures on the nozzle with a separation of 19 mm and their leads routed along shallow channels milled into the nozzle. The RTD and TEM leads are soldered to male connectors that plug into a female connector soldered to one end of an L-com male HD15 cable. This cable connects to a precision temperature controller (IXL Lightwave LDT 5948) capable of driving up to 5 A at 12 VDC, which is just under the 6 A maximum rating for the TEMs.

The nozzle–TEM–heatsink assembly is press fit into an insulating foam block that has been milled with complementary step edges to ensure the proper registration of the two halves during assembly. The assembled head slips into a rectangular Delrin sleeve with a kinematic aluminum support designed to properly and repeatably position it (not shown).

Thermocouples integrated into the heatsink record the temperature of the coolant entering and exiting. Their temperature difference is proportional to heat transferred to/from the coolant, and their average provides a reliable estimate of the temperature on the heatsink side of the TEMs. The coolant is a 20% propylene glycol–water mixture with a density of 1.03 g ml^−1^ and specific heat of ~4 J g^−1^ K^−1^. Hence, the heat transferred to/from the coolant can be estimated knowing the coolant flow rate and the measured temperature difference. For example, a flow rate of 3 ml s^−1^ translates to ~12 W K^−1^.

Although heat is transferred through only one surface of the nozzle, the magnitude of the gradient between opposite faces is small and inconsequential. This is because the thermal conductivity of aluminum is high, and the cross-sectional area of the internal features sandwiched between the two aluminum surfaces is large compared to their thickness. The 13-mm horizontal gap between TEMs on the upstream face of the nozzle is tailored to accommodate an off-axis (30°) 10× microscope objective capable of viewing the sample capillary with ~1μm spatial resolution (not shown). Despite this gap, the temperature profile remains quite uniform throughout most of the nozzle, as shown in Fig. 2(d).

To assess and optimize this system’s performance, a quantitative heat-transfer model for the TEM–nozzle assembly is presented.

### TEM heat transfer equation

Parameters used to characterize sources of heat and paths for heat flow are summarized in Fig. 3. Temperature control is achieved by driving current through the TEMs, which generates both Peltier heat ${\dot{Q}}_{P}$ and Joule ${\dot{Q}}_{J}$ heat. Peltier heat (W = VA) is equal to the product of current, i(A); Seebeck coefficient, $S\left(\text{V}{\;\text{K}}^{-1}\right)$; and junction temperature, $T_{J}(\text{K})$. Reversing the sign of the current reverses the direction of heat flow. The parasitic Joule heat $\left(W=i^{2}R\right)$ boosts the heating capacity of the TEMs but diminishes their cooling capacity.

Equation (1) from Chap. 3 of the 1994 CRC Handbook of Thermoelectrics^7^ is reproduced below, which identifies terms relevant for determining the net heat flow when operating a TEM in cooling mode, as follows:

(1) $$q_{c}=\left(\alpha_{p}-\alpha_{n}\right)iT_{1}-K\left(T_{2}-T_{1}\right)-i^{2}R/2,$$

where $q_{c}$ corresponds to the net heat flow from the object being cooled; the difference between $\alpha_{p}$ and $\alpha_{n}$ corresponds to S, the Seebeck coefficient; i is the current; $T_{1}$ corresponds to the absolute temperature of the cold junction; $T_{2}$ corresponds to the hot junction; K corresponds to the TEM thermal conductance; and R is the TEM resistance. The author acknowledges that the coefficients S, K, and R are temperature dependent and states “*Nevertheless, a reasonable estimation of the performance may be made using averaged parameters*.”’

Using Eq. (1) as a guide, Eq. (2) is written as a more general heat-transfer equation that conforms to the nomenclature in Fig. 3 and accounts for thermal conductance between the nozzle and ambient through the foam insulation,

(2) $$\dot{Q}=iST-G_{H,T}\left(T_{H}-T\right)-G_{A}\left(T_{A}-T\right)-i^{2}R_{T}/2,$$

where $\dot{Q}$ is the net heat extracted from the nozzle; i is the current; S is the Seebeck coefficient; T, $T_{H}$, and $T_{A}$ correspond to the nozzle, heatsink, and ambient temperatures, respectively; $G_{H,T}$ is the thermal conductance across the TEM and takes into account its temperature dependence; $G_{A}$ is the thermal conductance from nozzle to ambient through the foam insulation; and $R_{T}$ is the temperature-dependent TEM resistance.

Before solving Eq. (2), the temperature dependence of $G_{H,T}$ and $R_{T}$ needs to be properly accounted for. As a first-order approximation, it is assumed that the temperature dependence of the thermal conductance is linear according to the following equation:

(3) $$G_{H,T}=G_{H}\left(1+\gamma \left(\left\langle T_{TEM}\right\rangle -T_{0}\right)\right),$$

where $T_{0}$ is a reference temperature at which $G_{H,T}=G_{H}$ and $\left\langle T_{\text{TEM}}\right\rangle$ is the average temperature in the Bi_2_Te_3_ posts.

When current flows through the TEMs, the temperature profile across the Bi_2_Te_3_ semiconductor posts is approximated by

(4) $$T_{TEM}(x)=T+\left(\frac{T_{H}-T}{L}\right)x+\frac{i^{2}R_{T}}{12G_{H,T}}\cdot \frac{6\left(xL-x^{2}\right)}{L^{2}},$$

which represents the solution to the second-order differential equation for one-dimensional heat flow with an internal heat source, where L corresponds to the length of the posts. In the absence of Joule heating, the temperature profile inside the posts is linear. In the presence of Joule heating, the average temperature is elevated by a magnitude equal to the Joule heat divided by 12 times its thermal conductance. Since the temperatures on either side of the TEMs are pinned, the temperature profile with Joule heat is no longer linear and becomes parabolic and the average temperature in the posts is

(5) $$\left\langle T_{TEM}\right\rangle =\frac{\left(T+T_{H}\right)}{2}+\frac{i^{2}R_{T}}{12G_{H,T}}.$$

Independent characterization of the TEM temperature dependent resistance and fitting the experimental values with a quadratic polynomial in terms of $\left\langle T_{\text{TEM}}\right\rangle$ yields $R_{T}$,

(6) $$R_{T}=a+b\left\langle T_{TEM}\right\rangle +c{\left\langle T_{TEM}\right\rangle }^{2},$$

where a=-0.495Ω, $b=7.10\times 10^{-3}\;\Omega /\text{K}$, and $c=3.38\times 10^{-6}\;\Omega /{\text{K}}^{2}$. Since $R_{T}$ is a function of $\left\langle T_{TEM}\right\rangle$, Eq. (5) needs to be solved iteratively [see Fig. 4(b)].

### Steady-state performance

The experimentally determined current required to achieve a given nozzle temperature with these TEMs is plotted in Fig. 4(a). Also included are two predicted steady state $\left(\dot{Q}=0\right)$ curves generated from Eq. (2). The best-fit curve represents a three-parameter least-squares fit to the experimental data (γ, $G_{H}$, and $G_{A}$) with the Seebeck coefficient S for two Bi_2_Te_3_ TEMs operating in series set to 0.025 V K^−1^, as per the manufacturer specification. The best-fit parameter values are $\gamma =-0.0025\;\text{W}/{\text{K}}^{2}$, $G_{H}=0.248\;\text{W}/\text{K}$, and $G_{A}=0.027\;\text{W}/\text{K}$. The fit when γ is set to zero is significantly poorer, especially at high temperatures, which implies that modeling the temperature dependence of $G_{H}$ with a linear approximation is sufficient to accurately represent the experimental data. Due to current-induced Joule heating, the average temperature in the TEM posts, $\left\langle T_{\text{TEM}}\right\rangle$, calculated according to Eq. (5), is elevated above the midpoint between T and $T_{H}$. Also shown in Fig. 4(a) is ΔT per amp, the slope of the fitted curve, which defines the temperature sensitivity to current. Note that the optimal PID gain parameters for the lightwave temperature controller are influenced by the magnitude of -dT/di, as discussed in the section titled “*Dynamic performance*.”

How might a negative value for γ be explained? The phonon contribution to heat conductance has a negative temperature coefficient due to phonon–phonon scattering. If the electron/hole contribution to thermal conductance over the operating temperature range is saturated by its semiconductor doping level, its positive temperature coefficient could be overwhelmed by the phonon contribution. Indeed, the temperature-dependent TEM thermal conductivity reported in Riffat *et al*. is a decreasing function of temperature over most of the operating range.^8^

Figure 4(b) charts the resistance, $R_{T}$, and thermal conductance, $G_{H}$, and $G_{H,T}$, as a function of current given the best-fit γ. The increasing resistance with increasing temperature boosts Joule heating and thereby increases the TEM heating power. The decreasing TEM thermal conductance with increasing temperature reduces heat flow back to the heatsink and further increases the TEM heating power.

Note that Eq. (1) comes with the caveat that the Seebeck coefficient, thermal conductance, and resistance are all temperature dependent, but to varying degrees. In this analysis, the temperature dependence of $R_{T}$ in Eq. (2) is explicitly accounted for, while the temperature dependence of $G_{H}$ is assumed to be linear. However, the temperature dependence of S is not accounted for and is treated as a constant. Although this model for TEM performance reproduces the experimental observations with high fidelity, the best-fit values for γ, $G_{H}$, and $G_{A}$ should be viewed as approximate.

As discussed below, this model helps guide the specification of operating parameters tailored to achieve high performance while limiting the stress that leads to microcrack formation, propagation, and the ensuing degradation of TEM performance.

### Dynamic performance and temperature stability

The TEM current that maximizes the rate of heat transfer can be estimated as a function of temperature by taking the derivative of Eq. (2) and solving for current,

(7) $$i=\frac{ST}{R_{T}},$$

which for this system is ~4 A over the operating temperature range. However, when cooling at such high current, the temperature profile across the TEM posts, as defined by Eq. (4), exhibits a peak that rises above the high temperature junction. The Joule-heating enhanced temperature gradient along the length of the TEM post [see Eq. (4)] can act as a stress concentrator and accelerate the formation and/or propagation of microcracks. Although the maximum current specification for these TEMs is 6 A, it would not be prudent to operate them at that limit. When limiting the current to 3 A, the cooling rate is only marginally reduced, but the Joule heating-induced stress in the TEM posts is reduced significantly. Accordingly, with thermal cycling longevity, one of the principal goals of this effort, the operational current limits were set to ±3 A.

The time-dependent nozzle temperature T, heatsink temperature $T_{H}$, and slew rates dT/dt when transitioning between −20 and 130 °C are charted in Fig. 5(a). The reservoir was switched from LT to HT promptly after starting the up ramp, and from HT to LT promptly after starting the down ramp, as tracked by ${\text{T}}_{\text{H}}$. When heating, Joule heat adds to the Peltier heat and increases the slew rate. When cooling, Joule heat subtracts from the Peltier heat and decreases the slew rate. The heating slew rate achieved allows the full operating range of 150° to be traversed in under one minute. The cooling slew rate at high temperatures is also quite fast, but below room temperature slows below 1 K s^−1^. Note that the slew rate achievable is limited by the heat capacity of the nozzle and TEMs, which is estimated to be ~15 J K^−1^. Hence, the 3 °C per second peak heating slew rate corresponds to ~45 W heating, of which ~22 W is Peltier heat.

The temperature stability achieved at three different target temperatures is charted in Fig. 5(b). To achieve high temperature stability over the entire operating range, over which ΔT per amp varies by nearly an order of magnitude [as seen in Fig. 4(a)], different PID gain parameters were employed for the low-, moderate-, and high-temperature ranges. For example, the results obtained in Fig. 5(b) were obtained with the P and D gains at low temperature set 2.4 times greater than those employed at high temperature. The rms temperature fluctuations at low temperature when circulating LT coolant are ~5 mK. At high temperature, when circulating HT coolant, it rises to ~10 mK due to its periodic (~23 min), thermostat-controlled heating cycle. Near room temperature, the stability is within a few mK.

### TEM damage from thermal cycling

With 4 °C coolant circulating through the heatsink and the control temperature repeatedly ramped between −20 and 120 °C at a rate of nearly 1 K s^−1^, the low temperature slew rate perceptibly degraded after only a few tens of cycles. This degradation in performance was found to be due to a progressive increase in the TEM resistance, which increased parasitic Joule heating. When backing off the high temperature limit to 100 °C, the degradation continued but at a dramatically lower pace. It is well known that thermal cycling can be harmful to TEMs, but the rapid degradation observed was unexpected and required frequent TEM replacement. What is the origin of this degradation, and what can be done about it?

Several prior studies examined the deleterious effects of thermal cycling on TEMs. Hori *et al*. performed thermal cycling tests on TEMs with thermocouple posts of varying cross-sectional area.^9^ They concluded that performance loss was due to an increase in electrical resistance resulting from separation between the TEM posts and the solder. A 2009 study by Hatzikraniotis *et al*. performed thermal cycling experiments on Bi_2_Te_3_ TEMs and used scanning electron microscopy (SEM) to determine the source of degradation.^10^ The SEM images revealed the formation of cracks in the posts after thermal cycling. In a 2013 study, Barako *et al*. performed extensive thermal cycling tests on TEMs until they reached a point of critical failure.^11^ After 1000 thermal cycles, only a modest decrease in ZT, the TEM figure of merit, was recorded. However, at 10 000 cycles, ZT began to decrease rapidly until total device failure at 45 000 cycles. The thermal images of a failed TEM revealed cracks in the posts near the solder junction. They concluded that the damage to TEMs and subsequent loss in electrical conductivity and cooling performance are primarily caused by differences between the expansion coefficients of the posts and solder.

The number of thermal cycles tolerated before TEM degradation appears to vary wildly. How can these differences be rationalized? The thermal expansion coefficient for alumina is 8.1 × 10^−6^, which is small but not zero. The Bi_2_Te_3_ posts are rigidly anchored to alumina plates on either side via solder connections. A temperature difference across the TEM results in the differential thermal expansion of the alumina plates and will generate strain on the brittle Bi_2_Te_3_ posts in proportion to the dimension of the TEM, with the strain being greatest in the corners. When operating with a large temperature difference across the TEM, this strain can lead to the propagation of microcracks in the Bi_2_Te_3_ posts and a concomitant increase in electrical resistance. The TEMs used in the 2013 Barako study consisted of a 4 × 4 array of Bi_2_Te_3_ posts, and the largest temperature difference across their TEM was about 123 °C. The TEMs used in the temperature control head consist of 10 × 10 arrays of Bi_2_Te_3_ posts, and when operating at 120 °C, the temperature difference across the TEMs was about 116 °C. Assuming posts of the same dimension, the strain induced in the temperature control head setup would be 2.4 times that experienced in the Barako study. Hence, reducing the maximum temperature difference across the TEMs, especially when operating at high temperature, would be expected to slow TEM degradation when thermally cycled. To test this idea, an Oasis 160 water chiller was programmed to synchronously change its coolant temperature between 8 and 45 °C (maximum setpoint allowed) while executing the temperature ramp (T-ramp) sequence, thereby reducing the maximum temperature difference across the TEMs to about 75 °C. With this improvement, degradation in the TEM performance was slowed by approximately an order of magnitude but not eliminated. Note that the maximum strain in the TEMs with this improvement was still about 50% greater than that experienced in the 2013 Barako study. It follows that limiting the maximum temperature difference across the TEMs to 50 °C or less should further slow the degradation in TEM performance and may extend the usable TEM lifetime to thousands of cycles.

### Three-reservoir coolant circulation system

To reduce the maximum temperature difference experienced across the TEMs while thermal cycling, a coolant circulation system was developed which employs low temperature (LT), room temperature (RT), and high temperature (HT) reservoirs, three pinch valves to select the reservoir, and a peristaltic pump to circulate the selected coolant through the temperature control head (Fig. 6).

The source and return lines for all three reservoirs are routed through dual channel solenoid-controlled pinch valves (Valcor Engineering Corporation, SV23D, 12-V). The pinch valves for the HT and LT reservoirs are configured to be normally closed, whereas the pinch valve for the RT reservoir is configured to be normally open. Therefore, with no pinch valve activated, the system circulates RT coolant. The source and return lines beyond the pinch valves are combined in cross fittings that connect to the temperature control head and the peristaltic pump (Boxer 15QQ 4-roller pump head; Nema 17 Frame Stepper Motor; A2 Driver; 4.8 mm ID tubing). Peristaltic pumps function by rotating rollers over the tubing, compressing the tubing and pushing fluid through the line. The coolant flow rate is controlled by the rate of rotation, whose maximum in this setup is about 3 ml s^−1^. When pushing coolant through the head with positive pressure at high temperature, the silicone seal between the heatsink and TEM was found to be vulnerable to rupture and subsequent leakage. Hence, the plumbing was reversed so that the peristaltic pump draws coolant through the temperature control head rather than pushing through it, thereby minimizing the risk of coolant leaks in the head. The RT reservoir is used when operating the nozzle between 10 and 85 °C. When operating below 10 °C, both LT and RT pinch valves are activated, which circulates LT coolant; when operating above 85 °C, both RT and HT pinch valves are activated, which circulates HT coolant.

The peristaltic pump and pinch valves have no direct contact with the coolant. Consequently, their respective heat capacities have minimal influence on the temperature of the coolant flowing through the silicone tubing. Moreover, the heat capacity and thermal conductivity of the silicone tubing are low, which facilitate large, rapid temperature changes that minimize dead time when probing samples at different, wide-ranging temperatures. Switching between reservoirs dumps coolant at a different temperature into the currently selected reservoir and imposes an additional thermal load on that reservoir. Since the ~30-ml dead volume in the external coolant loop is small compared to the reservoir capacity, this thermal load is manageable. Switching the reservoir according to the target temperature allows us to maintain temperature differences across the TEMs at or below 45 °C when operating over the entire −20 to 130 °C temperature range.

The photographs of the temperature control apparatus are shown in Fig. 7. The temperature control head can be seen atop the lightwave temperature controller alongside the complete temperature control system, which consists of a high temperature (HT) hot water reservoir (InSinkErator^™^ HWT-00), a low temperature (LT) cold water reservoir (InSinkErator^™^ CWT-100), a room temperature (RT) reservoir, a peristaltic pump, three pinch valves that select the coolant source/return, and a networked Raspberry Pi computer programmed to operate the pump, pinch valves, and precision temperature controller. Simple modifications were made to both heater and chiller, which function as temperature-controlled coolant reservoirs with stainless steel tanks. Setting the temperature control on the heater reservoir (2.5 l) to its maximum produced a temperature that cycled between ~80 and 87 °C. An internal screw that controls the low temperature limit of the chiller reservoir (1.4 l) was adjusted to attain a minimum temperature that cycled between ~3 and 4.5 °C. The HT and LT reservoirs were plumbed with an overflow tube that empties into the RT reservoir (0.5 l), which accommodates the thermal expansion/contraction of the coolant when its temperature changes.

A headless Raspberry Pi 4 model B quad core single board computer running Linux is used to operate the temperature control system. The computer controls a HAT interconnected four-channel relay board (Docker Pi, Ltd., EP-0099) that is configured to switch 12 V power to the stepper-motor-controlled peristaltic pump and three solenoid-activated pinch valves. The computer also controls a HAT interconnected thermocouple board (Measurement Computing MCC 134) with two of its four channels assigned to record the temperature of the source and return ports on the heatsink. The speed of the peristaltic pump is controlled by a pulse width-modulation (PWM) signal generated by the Raspberry pi but is constrained to operate between 20% and 100% of full speed, with full speed set by an external resistor to achieve a coolant flow rate of ~3 ml s^−1^. The Raspberry Pi communicates with the Lightwave temperature controller via a StarTech USB to RS232 adapter. Commands used to operate the temperature controller include turning the temperature control feedback on/off, reading the nozzle temperature, reading the TEM current and voltage, changing the set temperature, and changing PID parameters.

### TEM stress test

The three-reservoir water circulation system described here limits the maximum temperature difference between the nozzle and heatsink to less than 45 °C and should significantly curb the degradation in TEM performance during thermal cycling. The Tramp sequence used for the thermal cycling stress test entailed ramping the nozzle temperature between −10 and 120 °C in 1° steps and holding for a period of time at each extremum. A single repeat in the linear T-ramp sequence is charted in Fig. 8, in which the ramp is linear over most of the range but deviates from linearity when the cooling current saturates at its 3 A maximum.

For this stress test, a temperature control head was prepared with fresh TEMs whose series resistance prior to use was measured with an LCR meter (BK Precision 878B). The series resistance was remeasured after completing 250 T-ramp sequences, and again after another 250 T-ramp sequences. No increase in resistance was observed, which implies no degradation of the TEMs over 500 thermal cycles and confirms the hypothesis that minimizing the temperature difference across the TEMs during operation and limiting the current delivered to them can circumvent TEM degradation.

### X-ray scattering of water from −16 to 120 °C

To test the performance of the temperature control system, temperature-dependent x-ray scattering data were acquired from water circulating in a capillary on the time-resolved 14-ID-B BioCARS^12,13^ beamline at the Advanced Photon Source, Chicago, IL. The temperature controller was programmed to ramp the nozzle temperature between −16 and 120 °C at a rate of nearly 1 K s^−1^, holding for a period of time at each extremum, and repeating the sequence three times. The sample positioning and circulation setup used for these measurements will be described in a future paper.

When 12-keV x rays pass through water in the capillary, a small fraction are elastically scattered at angles that correspond to pairwise distances between atoms and, hence, the molecular structure of liquid water. One-dimensional scattering curves generated from azimuthal averages of 2D scattering images, which span both Small- and Wide-Angle X-ray Scattering regions (SAXS and WAXS), are charted as a function of temperature in Fig. 9(a). The scattering amplitudes were divided by the temperature-dependent density of water to ensure that all curves are on the same absolute scale, i.e., the same number of electrons within the volume probed by the x-ray beam.

Water is an unusual fluid with a maximum density at 4 °C, a minimum in its isothermal compressibility around 46 °C and a maximum in its speed of sound around 74 °C, all of which presumably have structural origins relating to hydrogen bonding networks. Hence, its scattering pattern would be expected to reflect structural changes occurring over the entire temperature range examined, with at least four basis scattering patterns required to properly account for the temperature dependent scattering. Indeed, singular value decomposition of the data in Fig. 9(a) suggests that four vectors are necessary but sufficient to reproduce the temperature dependence observed. Note that the singular value for the fifth vector is very close to the noise floor in the normalized singular values chart in Fig. 9(b) and can be neglected. The noise floor is near the 10^−4^ level thanks to the high signal-to-noise ratio of the scattering data. The first four basis scattering vectors are charted in Fig. 9(c) and their temperature dependent amplitudes in Fig. 9(d). The first basis vector (blue) corresponds to the average scattering profile whose amplitude is nearly constant across the entire temperature range. The second basis vector (green) is strongly correlated with temperature and can be used as a non-invasive probe of temperature when performing T-ramp and T-jump measurements. For example, the temperature of the capillary contents lags behind the RTD temperature when ramping due to its heat capacity and finite rate of thermal conduction. This non-invasive probe of the temperature of the capillary contents allows us to characterize the lag time, from which the temperature of the capillary contents can be predicted with high precision given the time-dependent RTD temperature. Moreover, this non-invasive probe can be used to characterize the magnitude and duration of a laser-induced T-jump when pursuing time-resolved T-jump studies of biomolecules, which evolve on times scales far too fast to be probed with thermocouples or RTDs.

Note also the temperature dependent scattering amplitude in the low-q SAXS region of Fig. 9(a), which correlates with density fluctuations, whose minimum corresponds to the minimum in the isothermal compressibility of water. The third (red) and fourth (cyan) UT vectors in Fig. 9(c) are weaker with both exhibiting a SAXS feature that corresponds to the gradual appearance of long-range order in the hydrogen bonding network when super cooled.

Clearly, the water scattering data in Fig. 9 are rich in structural information regarding hydrogen bonding networks; however, its detailed interpretation is beyond the scope of this work.

## SUMMARY

The temperature control system reported here is capable of rapidly changing and precisely controlling the temperature of samples flowing through a capillary to 10 mK rms over a range spanning −20 to 130 °C. Key to its performance is a lightweight, clam-shell design aluminum nozzle that efficiently exchanges heat with the inserted capillary and achieves a uniform temperature profile spanning ~60 mm. Linear temperature ramps with a slew rate of 1 K s^−1^ can be achieved over most of the operational range, although parasitic Joule heating slows the slew rate when approaching subzero temperatures. Laminar helium flow over the capillary prevents condensation when cooling below the dew point, and the small volume being cooled minimizes the risk of freezing when supercooled. The temperature control head is compact with its aperture customizable depending on the application.

A relatively simple heat transfer equation reproduces the experimentally determined temperature vs current curve with high fidelity. This equation proved helpful when defining operational constraints that optimize performance while minimizing the risk of harm to the TEMs. For example, by selecting the coolant reservoir best suited for the target temperature (LT, RT, or HT), the temperature difference across the TEMs is limited to less than 45 °C, thereby limiting the strain suffered by brittle Bi_2_Te_3_ posts sandwiched between the TEM plates. Indeed, a temperature cycling stress test involving 500 thermal cycles between −10 and 120 °C with the operating current limited to ±3 A showed no hint of performance degradation, which validates this approach.

Although this temperature control system was developed to pursue temperature-dependent, time-resolved x-ray scattering studies of biomolecules flowing through a capillary, the openings in the nozzle housing can be tailored for use in spectroscopic applications as well. The use of LT, RT, and HT coolant reservoirs not only ensure temperature cycling longevity but also boosts the slew rate achievable, thereby reducing dead time when making measurements over a series of wide-ranging target temperatures.

## Figures and Tables

**FIG. 1.**
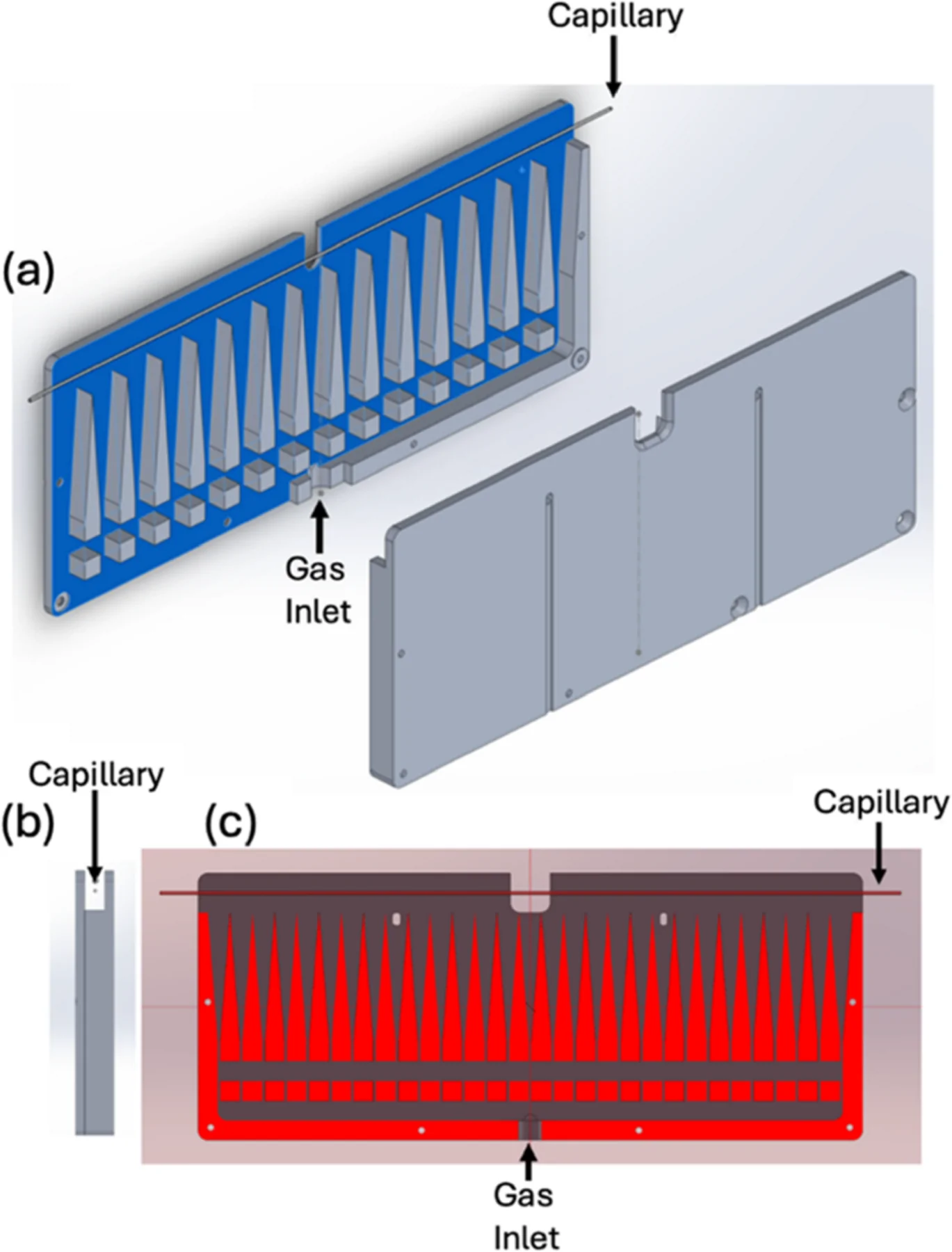
The aluminum nozzle has outer dimensions of 27 × 67 × 3.8 mm and weighs ~12 g. (a) The nozzle consists of two nearly identical parts whose array of 2-mm tall features are fabricated on a CNC milling machine using a flat-bottom 2-mm mill bit (depth of cut indicated by blue region). The two halves include alignment cylinders that snap together like LEGO^™^ pieces and are secured to each other with six 000–120 screws (three on each side), producing a 2-mm wide, 67-mm long slot through which dry gas flows and into which a capillary is inserted. Appropriately sized apertures are machined into each side of the nozzle to allow the passage of laser and/or x-ray beams through an inserted capillary. (b) Side view of slot with 0.32 mm OD capillary located in its center. (c) Cutaway view of the assembled nozzle. The structures machined into each half are offset relative to each other and, after assembly, produce two sets of 29 precisely spaced 2-mm long, 2-mm deep, 0.25-mm wide channels through which dry gas flows. The gas enters through the bottom opening (dark gray region) and flows between the red obstructions, which correspond to features contacting front and back faces of the nozzle. RTDs inserted into the oval slots are used to measure the temperature of the gas flowing through the nozzle.

**FIG. 2.**
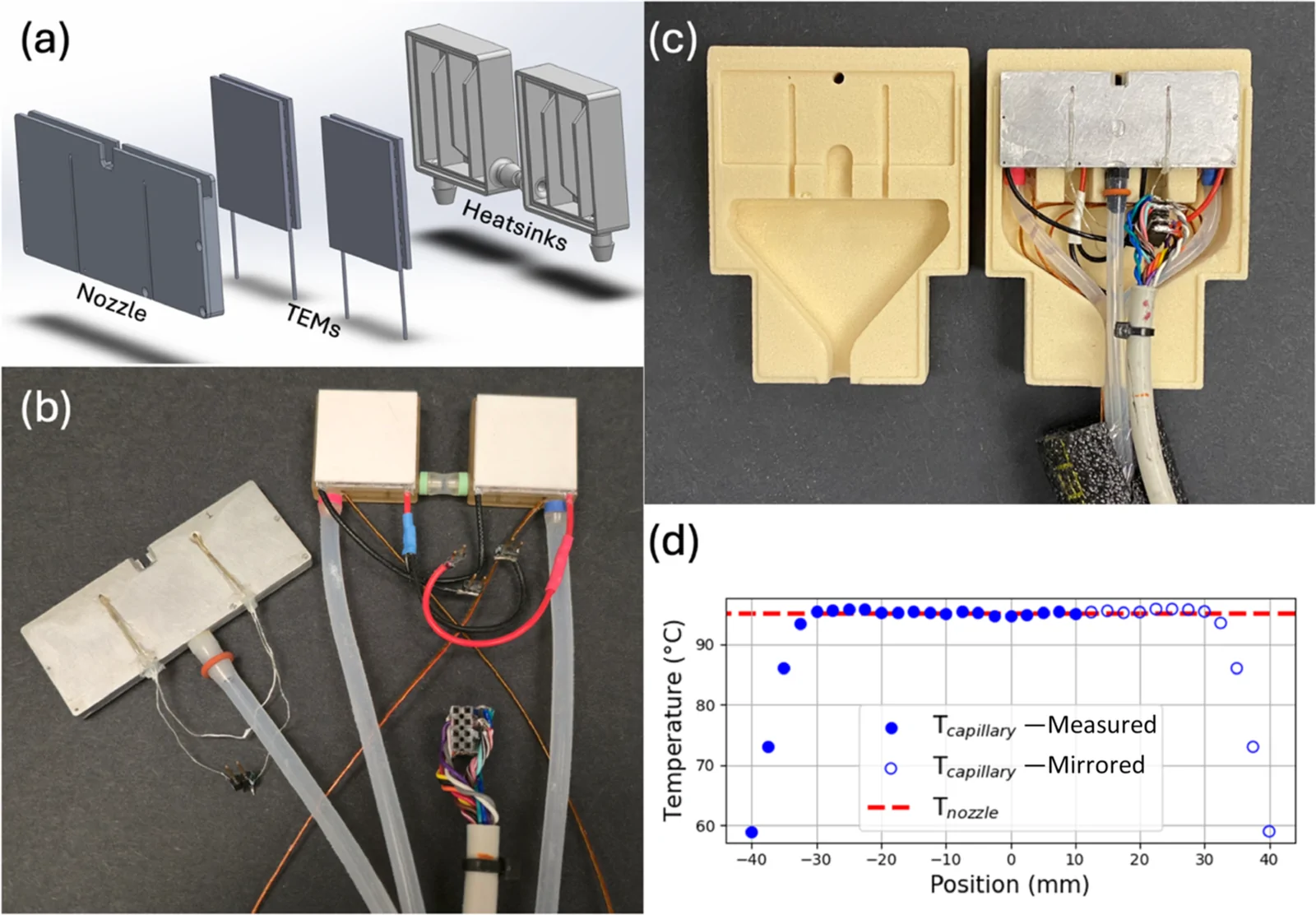
Temperature control head. (a) Exploded drawings of mirrored heatsinks, TEMs, and assembled clamshell nozzle. (b) A photograph of components housed inside the temperature control head. (c) A photograph of head showing separated halves of the insulating clam shell foam block with cutouts to accommodate the heatsink/TEM/nozzle assembly and plumbing/electrical connections. (d) Temperature profile recorded for a water-filled, 0.66 mm OD, fused silica capillary with the nozzle set to 95 °C (dashed red line). The nozzle boundaries are located at −33 and 33 mm. Position-dependent temperatures were measured (filled circles) by translating a small thermocouple through a water-filled capillary at 2.5 mm intervals starting from outside of the foam block housing (−40 mm) to 10 mm beyond the nozzle center. The open circles represent values mirrored relative to center as the profile is expected to be symmetric.

**FIG. 3.**
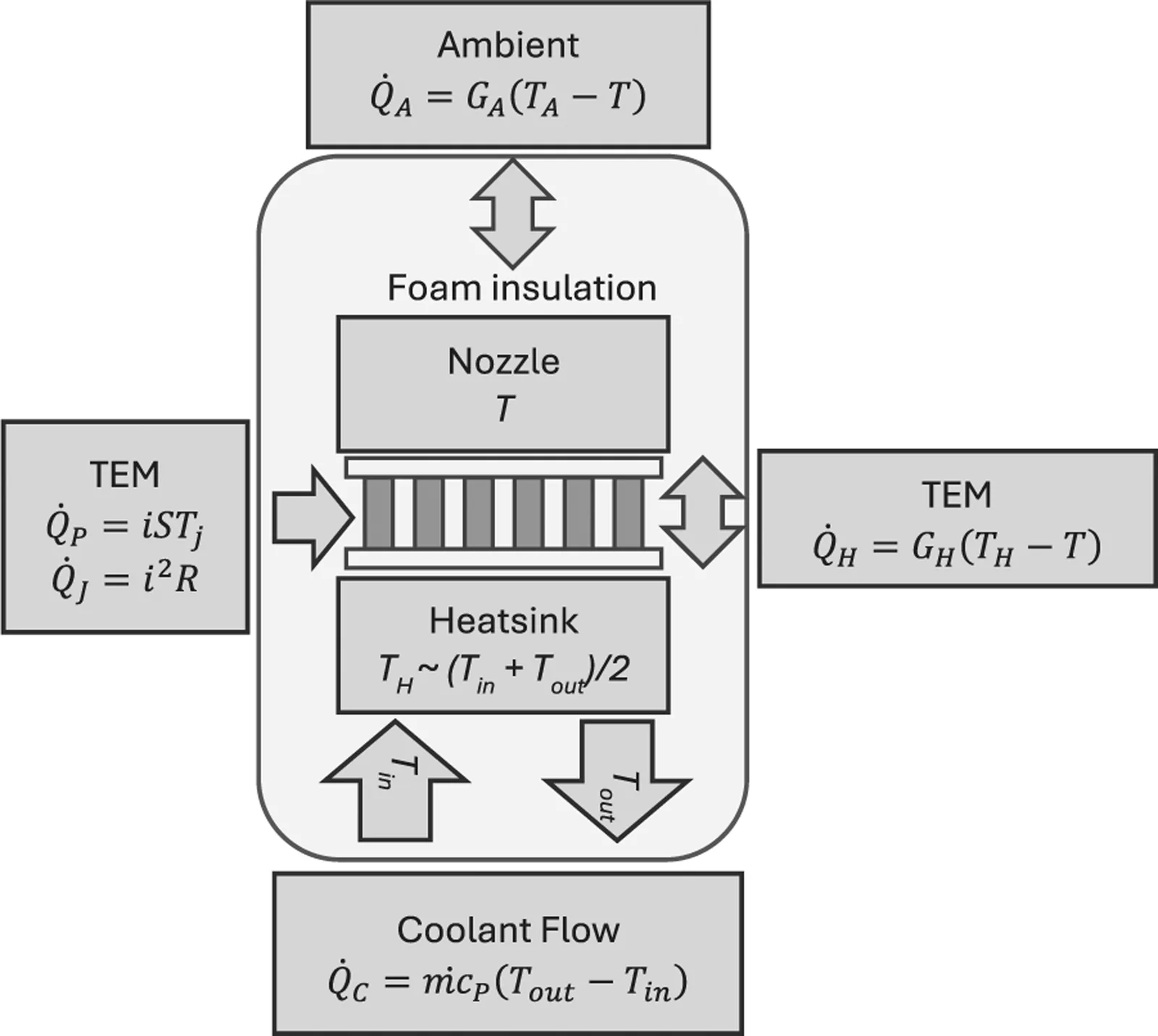
Block diagram indicating heat sources and heat flow. Heat sources include Peltier heat $\left({\dot{Q}}_{P}\right)$ and Joule heat $\left({\dot{Q}}_{J}\right)$. Heat flow from nozzle to ambient $\left({\dot{Q}}_{A}\right)$ and from nozzle to heatsink $\left({\dot{Q}}_{H}\right)$ is proportional to their temperature difference times their respective thermal conductance ($G_{A}$ and $G_{H}$). The coolant flowing through the heatsink extracts heat $\left({\dot{Q}}_{J}\right)$, whose magnitude is proportional to the temperature difference between coolant flowing in and out of the heatsink with the proportionality constant being a product of the coolant specific heat $\left[c_{P}\left(\text{J}{\;\text{g}}^{-1}{\;\text{K}}^{-1}\right)\right]$ and flow rate $\left[\dot{m}\left(\text{g}{\;\text{s}}^{-1}\right)\right]$.

**FIG. 4.**
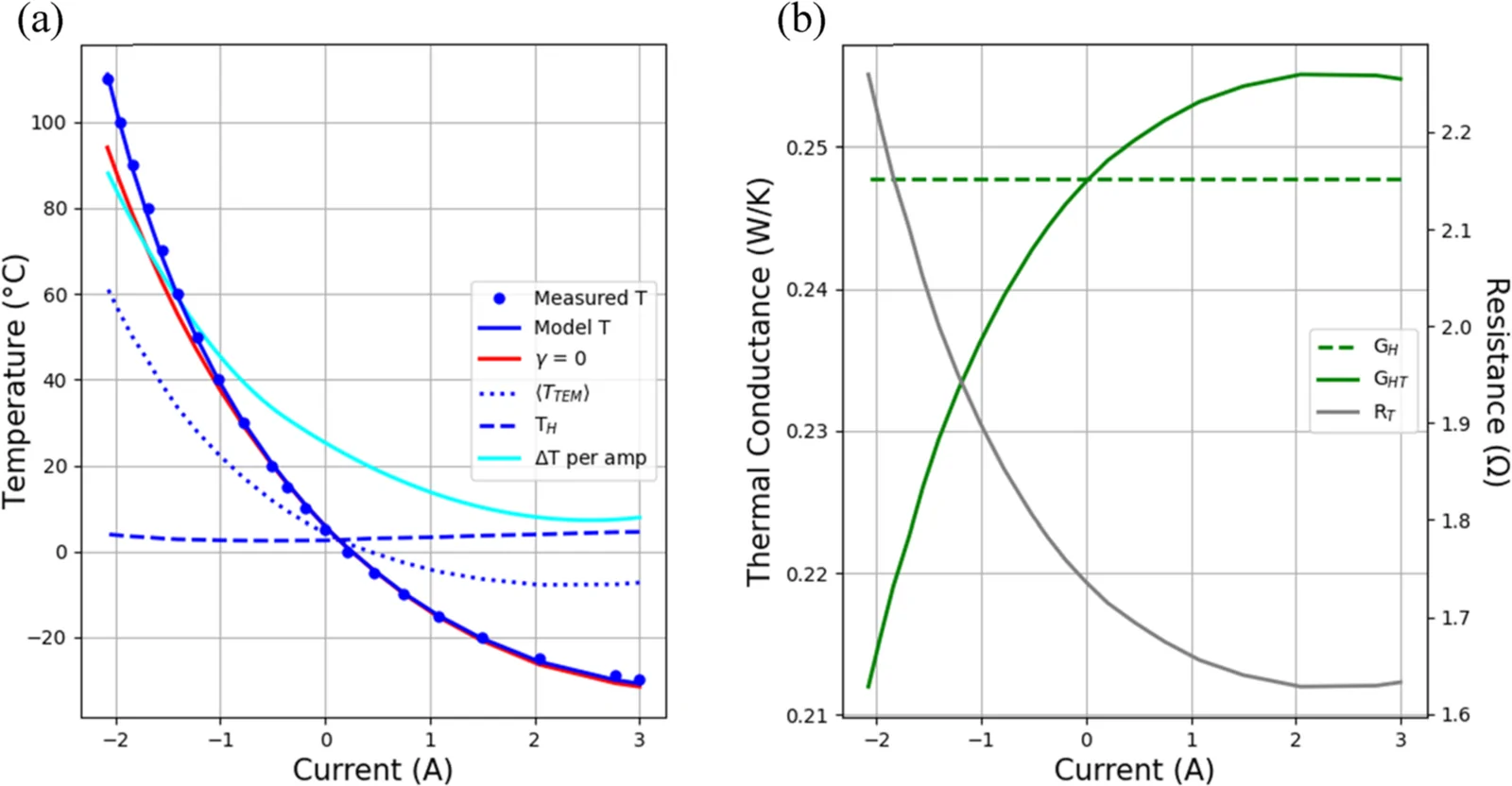
Measured and predicted steady-state parameters vs applied current. (a) Experimentally determined nozzle temperatures T as a function of TEM current (blue dots) are well described by Eq. (2) (blue curve). Also shown is Eq. (2) with γ set to zero (red curve). For reference, $T_{H}$ (blue dashed curve) and $\left\langle T_{\text{TEM}}\right\rangle$ (blue dotted curve) are shown. The negative slope of T (cyan curve), which is plotted in terms of ΔT per amp, varies by nearly an order of magnitude over this temperature range. (b) Estimated $R_{T}$ (gray curve), $G_{H}$ (green dashed line), and $G_{H,T}$ (green curve) as a function of current with optimized γ.

**FIG. 5.**
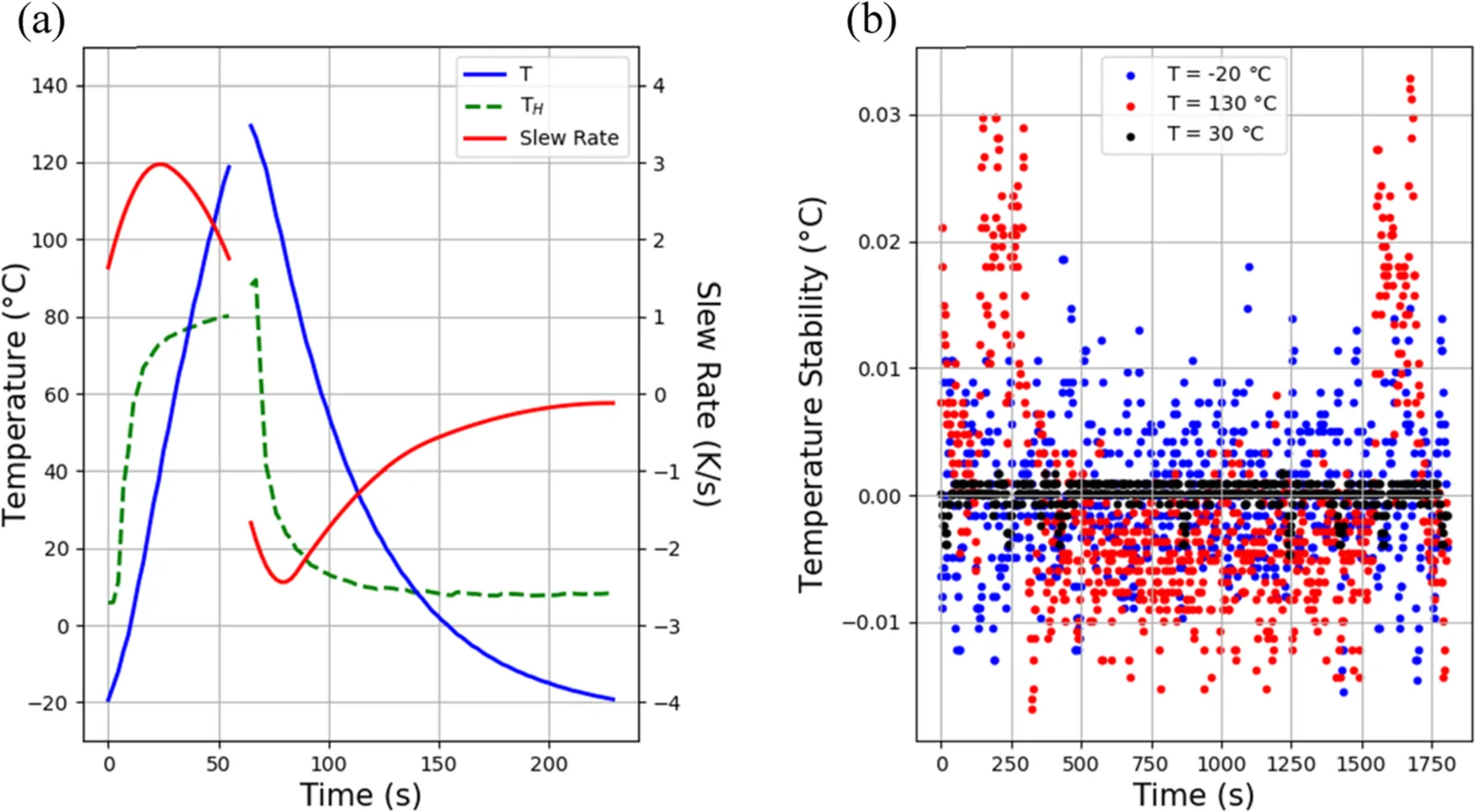
Dynamic performance of temperature control system. (a) Time-dependent nozzle temperature T (blue curve), heatsink temperature $T_{H}$ (green dashed curve), and slew rate (red curve) recorded when transitioning between minimum and maximum operating temperatures with the current limited to ±3 A. (b) Long-term temperature stability recorded at 130 °C (red), 30 °C (black), and −20 °C (blue).

**FIG. 6.**
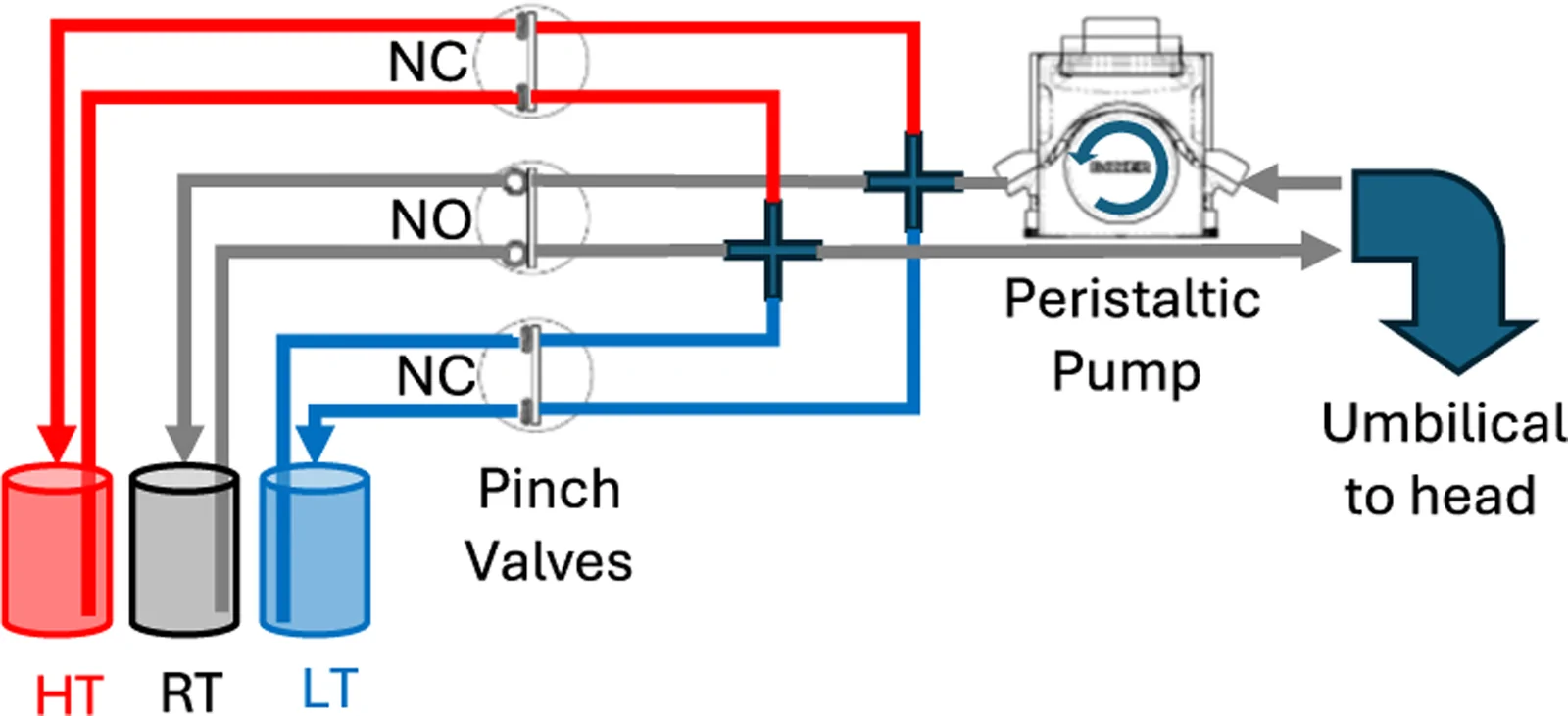
Schematic diagram of coolant circulation system. Source and return lines from LT (blue), RT (gray), and HT (red) reservoirs are connected through pinch valves to cross fittings that connect to the umbilical connected to the temperature control head (source) and the peristaltic pump (return).

**FIG. 7.**
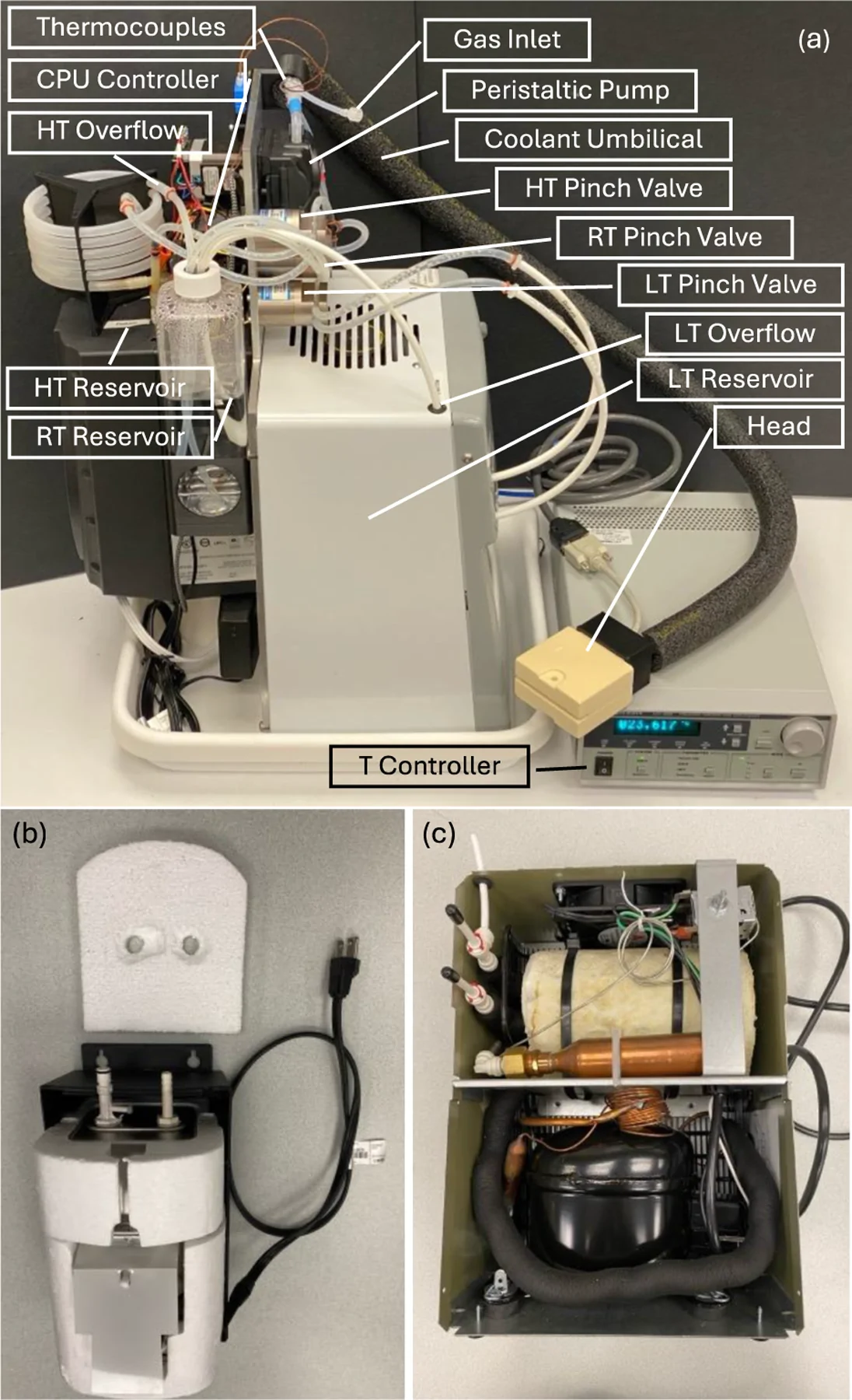
Temperature control apparatus. (a) The side view shows the high-temperature HT, room-temperature RT, and low-temperature LT coolant reservoirs, three cylindrical dual pinch valves that control flow through source and return lines, a peristaltic pump that controls the coolant flow rate, an insulated umbilical that delivers coolant to the remote temperature control head, and a high precision temperature controller. The cross fittings connect source and return lines from the three reservoirs to the external coolant loop. The HT overflow passes through a cooling coil enroute to the RT reservoir. The computer control system, which is housed in a 3D-printed fan-cooled enclosure located below the peristaltic pump, switches power to the stepper motor and pinch valves, controls the high precision temperature controller, and reads up to four thermocouple sensors. (b) Internal modifications to the water heater include the following: (1) removing a plastic assembly mounted on the top of the tank (not shown), (2) sealing the side port on the top left fitting, and (3) inserting a block of Styrofoam carved to fit in the empty space. (c) Internal modifications to the water chiller include the following: (1) disconnecting the copper water hammer cylinder from the water chiller tank, (2) drilling a hole in the top of the housing, (3) routing an overflow tube through the hole to the “T” fitting connected to the insulated stainless-steel tank.

**FIG. 8.**
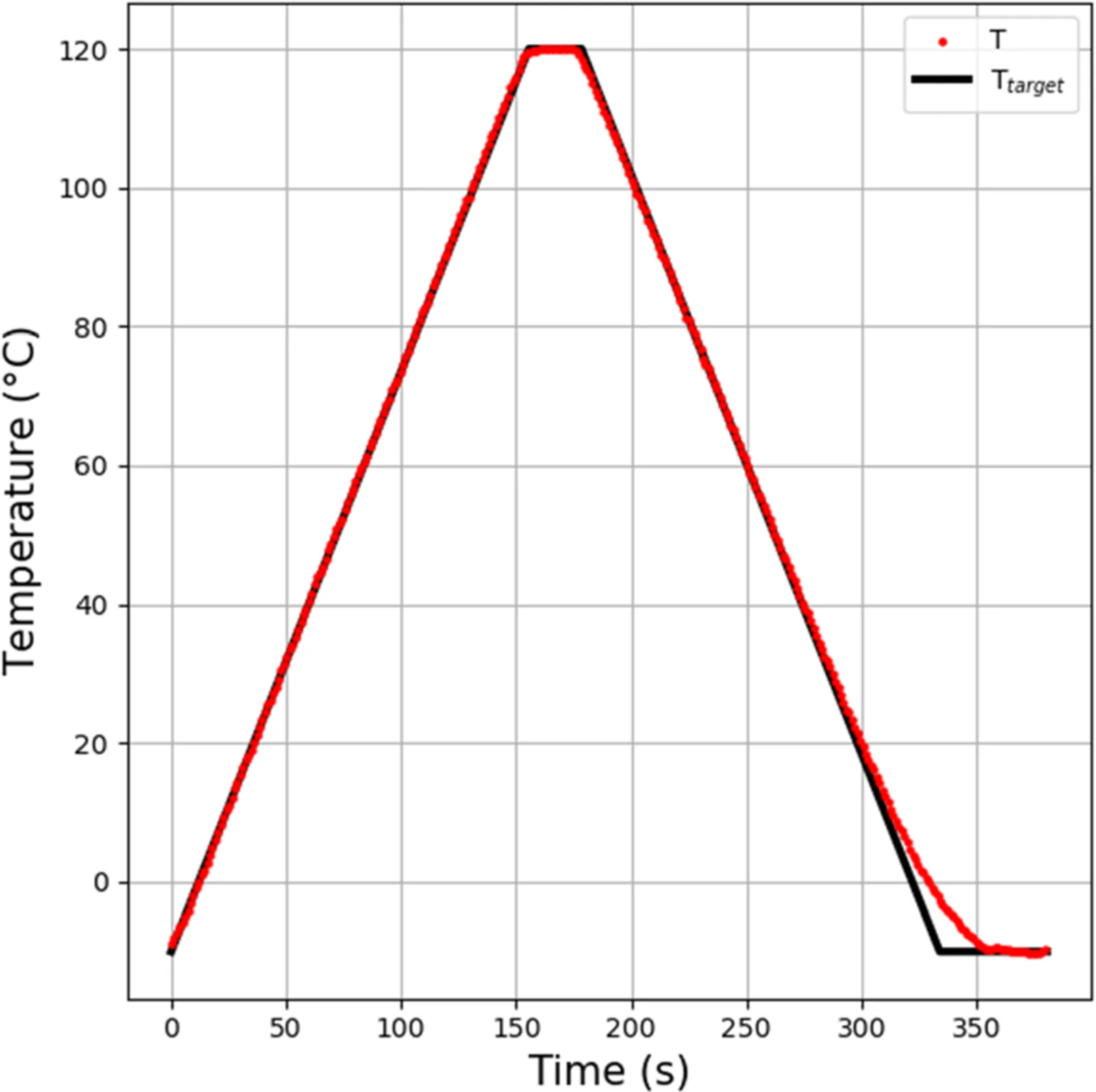
Single repeat of a T-ramp sequence with measured temperature (red dots) and target temperature (black line).

**FIG. 9.**
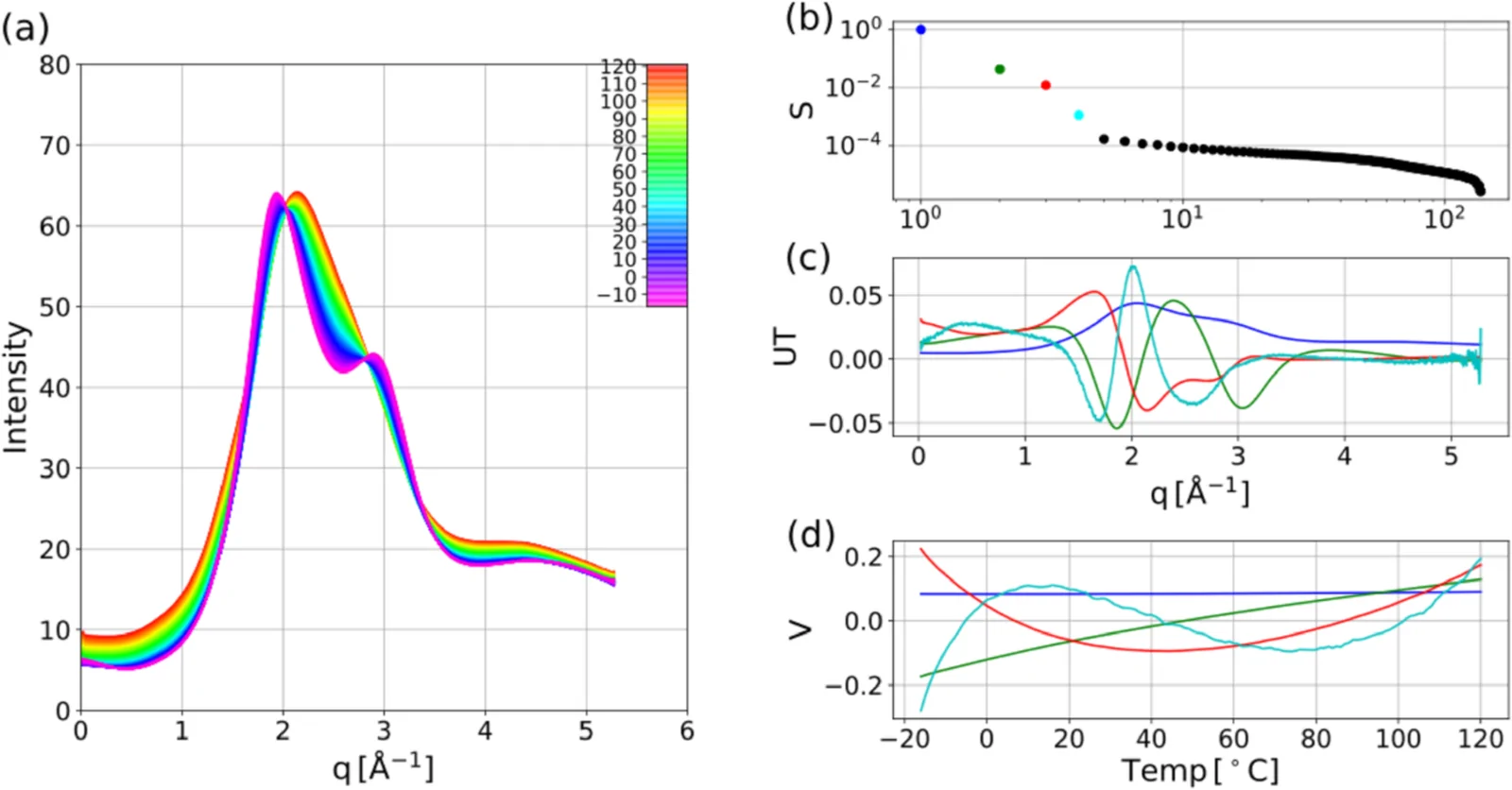
Temperature-dependent x-ray scattering of water from −16 to 120 °C. (a) The scattering curves are color-coded according to temperature and charted in 1 °C steps. (b) Normalized singular values S from singular value decomposition with first four singular values color coded. (c) First four basis scattering vectors (UT) from singular value decomposition. (d) Temperature-dependent amplitudes of first four basis vectors (V).

## Data Availability

The data that support the findings of this study are available from the corresponding author upon reasonable request.
